# Healthcare utilization, costs, and productivity losses in treatment-resistant depression in Finland – a matched cohort study

**DOI:** 10.1186/s12888-022-04115-7

**Published:** 2022-07-19

**Authors:** Heidi Taipale, Markku Lähteenvuo, Antti Tanskanen, Saara Huoponen, Saara Rannanpää, Jari Tiihonen

**Affiliations:** 1 Department of Forensic Psychiatry, University of Eastern Finland, Niuvanniemi Hospital, Niuvankuja 65, 70240 Kuopio, Finland; 2 Department of Clinical Neuroscience, Karolinska Institutet, Berzelius väg 3, 171 77 Stockholm, Sweden; 3 Center for Psychiatry Research, Department of Clinical Neuroscience, Karolinska Institutet, & Stockholm Health Care Services, Region Stockholm, Norra Stationsgatan 69, 11364 Stockholm, Sweden; 4 School of Pharmacy, University of Eastern Finland, Yliopistonranta 1, 70210 Kuopio, Finland; 5 Janssen Cilag, PL 15, 02621 Espoo, Finland

**Keywords:** Economic burden, Treatment-resistant depression, Major depressive disorder, Antidepressant, Costs

## Abstract

**Background:**

Due to its relatively high prevalence and recurrent nature, depression causes a major burden on healthcare systems, societies and individuals. Our objective was to investigate healthcare resource utilization and costs associated with treatment-resistant depression (TRD) compared with non-treatment-resistant depression in Finland.

**Methods:**

Of all patients aged 16–65 years and diagnosed with depression in Finland during 2004–2016, persons with TRD (*N* = 15,405) were identified from nationwide registers and matched 1:1 with comparison persons with depression who initiated antidepressant use but did not have TRD at the time of matching. TRD was defined as initiation of a third treatment trial after having failed two pharmacological treatment trials. Follow-up period covered 5 years after TRD or corresponding matching date (until end of 2018). Health care resource utilization was studied with negative binomial regression and costs of TRD (per patient per year) with generalized estimating equations, by adjusting for baseline costs, comorbidity and baseline severity of depression.

**Results:**

Persons with TRD (mean age 38.7, SD 13.1, 60.0% women) had more health care utilization and work disability (sick leaves and disability pensions), adjusted incidence rate ratio for work disability days was 1.72 (95% CI 1.64–1.80). This resulted in 1.9-fold higher total costs for persons with TRD (15,907 versus 8335 EUR), adjusted mean difference 7572 (95% CI 7215–7929) EUR per patient per year, higher productivity losses (due to sick leaves and disability pensions, mean difference 5296, 95% CI 5042–5550), and direct healthcare costs (2003, 95% CI 1853–2151) compared with non-TRD patients. Mean difference was the highest during the first year after TRD (total costs difference 11,760, 95% CI 11,314–12,206) and the difference decreased gradually after that.

**Conclusion:**

Treatment-resistant depression is associated with about two-fold cost burden compared with non-treatment-resistant depression.

**Supplementary Information:**

The online version contains supplementary material available at 10.1186/s12888-022-04115-7.

## Introduction

Depressive disorders are one of the leading causes of disability worldwide and have prevailed as a leading cause for decades [1]. Due to a relatively high prevalence, recurrent course and negative impact on work ability, depressive disorders typically result into frequent and long sick leaves, [2] culminating into vast societal and personal cost burdens in the form of permanent work disability [3]. Permanent work disability, in the form of disability pension is also associated with lower quality of life and income [4].

Treatment-resistant depression (TRD) has been suggested to be a major contributor in the societal costs of depressive disorders [5]. The definition of TRD is based on having had two consequential pharmacological treatment trials, with adequate length and dose, to which the patient has not responded or not achieved symptom remission [6]. Some definitions of TRD also include psychotherapy but detailed data on psychotherapy is lacking from many data sources and thus, cannot be included. TRD has been associated with multiple adverse outcomes as compared to regular major depressive disorder, including higher risk of suicides, [7] all-cause mortality, [8, 9] substance use disorders, [10] early labor force exit, [11] and lower quality of life and greater functional and work impairment [12]. Higher risk of disability pension [11, 13] and more frequent healthcare resource utilization in TRD [14–17, (Brenner et. al.: Health care utilisation in treatment-resistant depression: a Swedish population-based cohort study, unpublished)] lead to a major cost burden both for the society and the individuals. Previous studies on the cost burden of TRD have been conducted mainly in the US, [14, 16, 17] although also some European studies exist [18]. Cost burden and distribution of direct versus indirect costs may vary considerably between different health, work labor and social security systems and thus, more studies are needed on costs associated with TRD.

We have previously reported that the incidence of TRD in Finnish patients is 11% within 2 years of the initiation of first pharmacological treatment [19]. Here we continue to characterize these TRD patients by studying their healthcare resource utilization and costs related to TRD, including direct costs and costs related to productivity loss, in comparison to matched patients with non-treatment-resistant depression during a five-year period. We also assessed the proportion of patients having income from work, as indicative of being employed before and after TRD. This is, to our knowledge, one of the first large scale and long-term studies on the economic burden of TRD in Europe.

## Methods

### Data sources

Finnish nationwide register-based data was utilized for this study. Registers can be linked through personal identification codes assigned for each resident at birth or immigration (i.e. people with residence permit are included in the registers). The Care Register for the Health Care includes all visits to hospitals (since 1972) and specialized outpatient care (since 1998), including data on admission and discharge dates and recorded discharge diagnoses and operational codes. It does not cover primary care nor private health care, such as occupational health care. For this reason, we utilized also registers on sickness absences and disability pensions. Sickness absences and disability pensions are covered by the Finnish sickness insurance system and thus, recorded in the administrative registers. Sickness absences were collected from the Social Insurance Institution of Finland and disability pensions from the Social Insurance Institution and the Finnish Centre for Pensions. All sick leaves of ≥14 days are registered and paid as a part of the social insurance scheme (regardless of whether person is employed or not). All employees aged 16–67 years of age who are not able to work due to sickness are eligible for sick leave and the same applies also for persons who are not employed. The sickness allowance is on average 70% of the previous earnings and it has no upper limit or ceiling. Sickness absences are recorded regardless of the care setting, i.e. include also those prescribed by primary care and private healthcare. Similarly, disability pensions are a part of the social insurance scheme and recorded from all levels of care. A person who has been disabled for ≥300 working days can apply for disability pension (either permanent or a fixed-term disability pension, so called rehabilitation subsidy) regardless of their employment status (i.e. also unemployed or otherwise economically inactive can apply). In the registers, sickness absences and disability pensions include beginning and end dates and diagnoses. The Finnish National Prescription Register includes data on all reimbursed prescription drug purchases from Finnish pharmacies for all residents regardless of the care setting or the prescriber. Drugs provided during hospital care are not recorded but prescriptions stated by physicians working in the hospitals are record. The data includes information on date of dispensing, the Anatomic Therapeutic Chemical (ATC) code, strength, package size, the number of packages dispensed and dispensed amount in Defined Daily Doses (DDDs).”

### Study population

We identified all persons diagnosed with depression (ICD-10 F32-F33) from nationwide Finnish registers, including inpatient and specialized outpatient care (The Care Register for the Health Care), sickness absence (all sick leaves > 2 weeks) and granted disability pensions (through registers of Social Insurance Institution and Finnish Centre for Pensions). Sick leaves and disability pensions included also those stated by primary care and occupational health care specialists, covering a wide range of patients treated on different levels of care. For each person, the first diagnosis of depression in the age of 16–65 years during years 2004–2016 was taken. Exclusions were made due to bipolar disorder (F30-F31), schizophrenia-spectrum disorder (F20-F29) and dementia (G30, F00-F03) when these conditions were recorded before the first depression diagnosis.

As the definition of TRD is based on medication use (supplemented with specialized health care information on neuromodulation treatments), antidepressant and other psychotropic medication use was derived from the Prescription register data, which includes reimbursed dispensing of prescribed medications, between the years 2003–2018. Antidepressants were identified as ATC (Anatomical Therapeutic Chemical)-code N06A. For each person, we identified the first antidepressant dispensing around the first depression diagnosis, and this first dispensing was assigned as the index date for TRD definition. First depression diagnosis needed to be registered within a time interval of 30 days before and up to 365 days after the index date. Inclusion of new users was conducted by inserting a 180 day washout period before the index date, meaning that persons using an antidepressant or defined potential add-on medications for depression (antipsychotics, lithium, lamotrigine, valproic acid or carbamazepine), or who were administered ECT (electroconvulsive therapy), rTMS (repetitive transcranial magnetic stimulation), tDCS (transcranial direct current stimulation) or ketamine infusion as registered in the inpatient or specialized outpatient care register during the washout period, were excluded. In Finland, these treatments are provided only in specialized health care settings. These new antidepressant users, with a registered diagnosis of depression formed the base cohort for this study (*N* = 177,144).

Medication dispensing data from the Prescription register was transformed to drug use periods with ‘From drug purchases to drug use periods’ (PRE2DUP) method. The method estimates when drug use started and ended based on dispensing dates, amounts dispensed and drug-specific parameters defining lower limit for daily dose in continuous use, and by taking into account on stockpiling, days spend in hospital care and personal regularity of use [20].

### TRD criteria

TRD criteria were defined to be met when initiation of a third sequential treatment trial for depression occurred, after two preceding adequate trials (including the index trial and a second trial). Adequate treatment trial was defined as lasting for at least 28 days according to PRE2DUP modelled drug use periods. After first treatment trial with antidepressant, second and third treatment trials could consist of either a new antidepressant treatment, antipsychotic (quetiapine ≥100 mg, risperidone, olanzapine, aripiprazole) or mood stabilizer (lithium, valproic acid, carbamazepine, lamotrigine), or series of ECT, rTMS, tDCS or ketamine infusion (referred to as “ECT”, since only ECT was observed in our dataset before TRD criteria were fulfilled). Within drug-class changes were also considered new trials. Fulfillment of TRD criteria were followed for 2 years since index date, with maximum allowed gap (non-use) being set to 56 days.

### Matching of comparators

We excluded persons who were already on disability pension (DP) at the date of TRD to observe persons in working life (and at risk of productivity loss). At the date when TRD criteria were met and within the cohort of persons with depression, each patient with TRD was matched with one comparator who did not have TRD or disability pension, had first or second antidepressant trial ongoing, and had similar age (±2 years), the same gender, calendar year of index date, and hospital district (eFig. 1). Person who later was defined as TRD could serve as a comparator until the date when TRD criteria were met. No matching comparator could be found for *N* = 1248 TRD patients who were excluded. These excluded TRD patients were somewhat younger, more often male and had longer follow-up time than included TRD patients (Supplementary Table 1). For the final study, *N* = 15,405 patients with TRD and *N* = 15,405 matched comparators were included.

### Outcomes

Persons with TRD and their comparators were followed from TRD date (corresponding matching date for comparators) for 5 years, but censoring for death, diagnosis of bipolar disorder/ schizophrenia-spectrum disorder, fulfillment of TRD criteria for comparators and end of data linkage (December 31, 2018). Healthcare resource utilization was defined as all-cause inpatient hospital days and specialized outpatient care visits. For cost analyses, hospital day and outpatient visit costs were calculated according to Finnish healthcare system unit costs which have been derived for research purposes and adjusted for regional price differences [21]. Drug costs were calculated from costs of psychotropic medication dispensings as total costs recorded in the Prescription register data (mood stabilizers N03A, antipsychotics N05A, benzodiazepines and related drugs N05BA, N05CD and N05CF, and antidepressants N06A, other drug costs were not available). Psychotherapy costs covered costs paid by Social Insurance Institution, of state-funded psychotherapies and based on direct reimbursements paid to the healthcare provider. Hospital care, outpatient visits, medications and psychotherapies formed direct costs, whereas productivity losses covered work disability days, derived as days of sickness absence (SA) and/or disability pension (DP). Productivity losses related costs were calculated as SA and DP days multiplied by the average gross monthly salary across all sectors [22]. All costs were real-valued to Euros (€) in 2020 with the price index of public expenditure [23]. Costs were calculated for each consecutive year during 5 years, as per patient per year (PPY), by censoring persons not contributing to the study anymore.

Being employed was measured as having any income from work (yes/no), as derived from the Earnings- and Accrual register, maintained by the Finnish Centre for Pensions. The register is compiled based on calendar years, and year of TRD date was assigned as year zero. Development of the proportion of the cohort who were employed was followed for 4 years before and 4 years after year zero. The longitudinal association between TRD status and employment was assessed in two parts, during the years before and after TRD, year zero being included in the latter part.

### Analyses

Descriptive statistics were calculated with proportions, means with 95% confidence intervals and compared with chi squared tests and t-tests with parametric bootstrap procedure. Healthcare resource utilization and indirect resource utilization were analyzed by negative binomial regression models and reported as adjusted Incidence Rate Ratios (IRR) with 95% confidence intervals, with matched non-TRD patients as a reference. The models took into account follow-up time and were adjusted for baseline costs (defined as total costs during 1 year before index antidepressant dispensing), index period costs (defined as total costs during time period between index antidepressant dispensing and TRD date/ corresponding matching date, varying between matched pairs from 2 months up to 2 years but being equal within the matched pair), Charlson’s comorbidity index, and baseline severity of depression. Charlson’s comorbidity score [24] was calculated based on in- and specialized outpatient care diagnoses recorded during the last 5 years before the index date. Score of zero is assigned to persons who do not have any of the comorbidities recorded. Severity of depression at first diagnosis was categorized as mild (ICD-10: F32.0, F32.8, F32.9, F33.0, F33.8, F33.9), moderate (ICD-10: F32.1, F33.1), severe (ICD-10: F32.2– F32.3, F33.2–F33.3), or unknown (for persons who had their diagnosis recorded only with three characters in the register source used and for whom severity level could not be determined).

Costs were compared with Generalized Estimating Equations (GEE) models, specifying Poisson distribution and log link, between TRD and comparators. Models were constructed separately for total costs, direct healthcare costs and productivity losses. Cost analyses were adjusted for baseline and index period total costs, Charlson’s Comorbidity Index, and baseline severity of depression. Costs are reported as adjusted means for persons with TRD, compared with non-TRD comparators, as mean costs per patient per year (PPY). Costs for years 1–5 after TRD were analyzed similarly as adjusted GEE models with year interaction and expressed as adjusted mean costs with 95% confidence intervals. Subgroup analyses were conducted among patients with severe depression recorded before TRD date/ matching date, by additionally adjusting for matching factors (as matched design was not retained, i.e. age, gender, calendar year and hospital district). Sensitivity analyses for the main analyses were conducted by censoring both members of a TRD/ non-TRD matched pair when one member of the pair was censored, resulting into equal follow-up times. Finally, we conducted sensitivity analyses by matching non-TRD comparators (1:1) to those patients who were defined as TRD during 2 years follow-up at initiation of index antidepressant and followed them 5 years beginning from index antidepressant.

GEE logistic regression model was applied to employment analyses, by adjusting for Charlson’s Comorbidity Index and baseline severity of depression. Unstructured correlation option was used due to the unbalanced nature of data, where imbalance was caused by the varying number of persons included in each timepoint due to exclusion/ censoring. Data management was conducted with SAS version 9.4 (SAS Institute, Inc., Cary, NC, USA) and statistical analyses with STATA 17.0.

## Results

Persons with TRD were of mean 38.7 years old (SD 13.1) and 60.0% were women (Table 1). Comparators with non-TRD depression who had their first or second antidepressant trial were similar to persons with TRD in terms of matching factors, but had somewhat milder initial severity of depression (e.g. 11.0% had severe depression compared with 17.5% of persons with TRD). Baseline costs, measured as 1 year before index antidepressant dispensing, were relatively similar between persons with TRD and comparators, and some costs, such as hospital care costs were higher among comparators whereas persons with TRD had somewhat more SA days and associated costs. During the index period (mean duration 8 months), persons with TRD had higher costs than comparators.

**Table 1:** Baseline sociodemographic and clinical characteristics of the matched groups, and costs and resource utilization at baseline (one year before index antidepressant) and during index period (between index antidepressant and treatment-resistant depression, TRD or corresponding matching date for non-TRD persons). Comparators matched by age, sex, hospital district and calendar year of index date

	Non-TRD *N* = 15,405	TRD *N* = 15,405	*p*-value
Age at index date, mean ± SD (median), years	38.7 ± 13.1 (39)	38.7 ± 13.1 (39)	0.9411
Age categories, % (n)			0.9705
16–24	20.6 (3177)	20.7 (3189)	
25–34	22.2 (3412)	22.0 (3393)	
35–44	20.8 (3199)	20.7 (3188)	
45–54	22.7 (3503)	23.0 (3547)	
55–65	13.7 (2114)	13.6 (2088)	
Women, % (n)	60.0 (9242)	60.0 (9242)	1.000
Year of index date, % (n)			1.000
2004–2007	37.4 (5766)	37.4 (5766)	
2008–2011	32.6 (5022)	32.6 (5022)	
2012–2016	30.0 (4617)	30.0 (4617)	
Severity of depression, % (n)			<.0001
Mild	21.4 (3293)	18.7 (2884)	
Moderate	28.8 (4433)	27.0 (4152)	
Severe	11.0 (1692)	17.5 (2696)	
Unknown	38.9 (5987)	36.8 (5673)	
Charlson’s Comorbidity Index, mean ± SD (median)	0.01 ± 0.1 (0)	0.11 ± 0.4 (0)	<.0001
Follow-up time, mean ± SD (median), days	1482.5 ± 583.8 (1826)	1519.3 ± 531.6 (1826)	<.0001
Reason for end of follow-up, % (n)			<.0001
Death	1.9 (290)	2.4 (373)	
Exclusion diagnosis	4.1 (633)	12.1 (1862)	
End of data linkage	18.8 (2896)	19.5 (3003)	
TRD diagnosed for non-TRD	9.5 (1460)	0.0 (0)	
Full 5 years	65.7 (10126)	66.0 (10167)	
**Baseline costs and health care utilization,** mean ± SD (median)			
Total costs	2972.3 ± 6980.7 (797)	2953.6 ± 6357.3 (634)	0.806
Total direct costs	1497.2 ± 5304.9 (266)	1361.3 ± 4349.9 (266)	0.015
Hospital care costs	810.0 ± 4772.0 (0)	692.8 ± 3748.1 (0)	0.017
Outpatient visit costs	655.7 ± 1647.1 (266)	630.3 ± 1524.2 (0)	0.175
Medication costs	19.6 ± 150.6 (0)	26.4 ± 170.6 (0)	<.0001
Psychotherapy costs	12.0 ± 168.6 (0)	11.8 ± 170.6 (0)	0.923
Sickness absence costs	1475.0 ± 3616.2 (0)	1592.3 ± 3878.7 (0)	0.007
Hospital days	1.4 ± 8.0 (0)	1.2 ± 6.3 (0)	0.019
Outpatient visits	2.5 ± 6.2 (1)	2.4 ± 5.7 (0)	0.172
Sickness absence days	14.6 ± 35.8 (0)	15.8 ± 38.4 (0)	0.004
**Index period costs and health care utilization,** mean ± SD (median)			
Duration of index period (from index AD to TRD/ matching)	261.3 ± 192.1 (223)	262.5 ± 153.8 (223)	0.548
Total costs	9584.8 ± 13,830.2 (4947)	16,776.9 ± 16,273.8 (12352)	<.0001
Total direct costs	3418.5 ± 9650.4 (803)	6659.5 ± 12,191.5 (2097)	<.0001
Hospital care costs	1470.0 ± 8362.3 (0)	3813.8 ± 10,764.9 (0)	<.0001
Outpatient visit costs	1659.1 ± 3206.6 (266)	2437.9 ± 3907.3 (1063)	<.0001
Medication costs	230.4 ± 382.0 (113)	337.2 ± 441.9 (201)	<.0001
Psychotherapy costs	59.0 ± 405.4 (0)	70.7 ± 417.2 (0)	0.010
Sickness absence costs	6166.3 ± 8131.5 (2931)	10,117.4 ± 9390.2 (8287)	<.0001
Hospital days	2.5 ± 14.0 (0)	6.4 ± 18.0 (0)	<.0001
Outpatient visits	6.2 ± 12.1 (1)	9.2 ± 14.7 (4)	<.0001
Sickness absence days	61.0 ± 80.5 (29)	100.1 ± 92.9 (82)	<.0001
**Total baseline and index period costs,** mean ± SD (median)	8282.0 ± 12,976.8 (4554)	13,179.3 ± 14,812.9 (8801)	<.0001

*p*-values from chi squared test for categorical variables and from t-test for continuous variables from a parametric bootstrap procedure

**Fig. 1:**
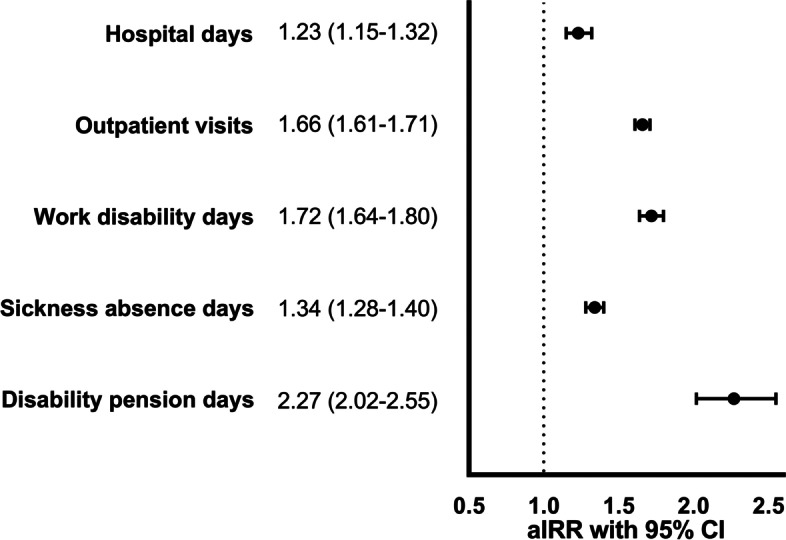
Health care resource utilization and indirect resource utilization measures during the 5 years follow-up. Adjusted Incidence Rate Ratios (IRR) comparing persons with treatment resistant depression (TRD) to matched non-TRD persons. Adjusted for: baseline and index period total health care costs, Charlson’s Comorbidity Index, follow-up time and baseline severity of depression

**Fig. 2:**
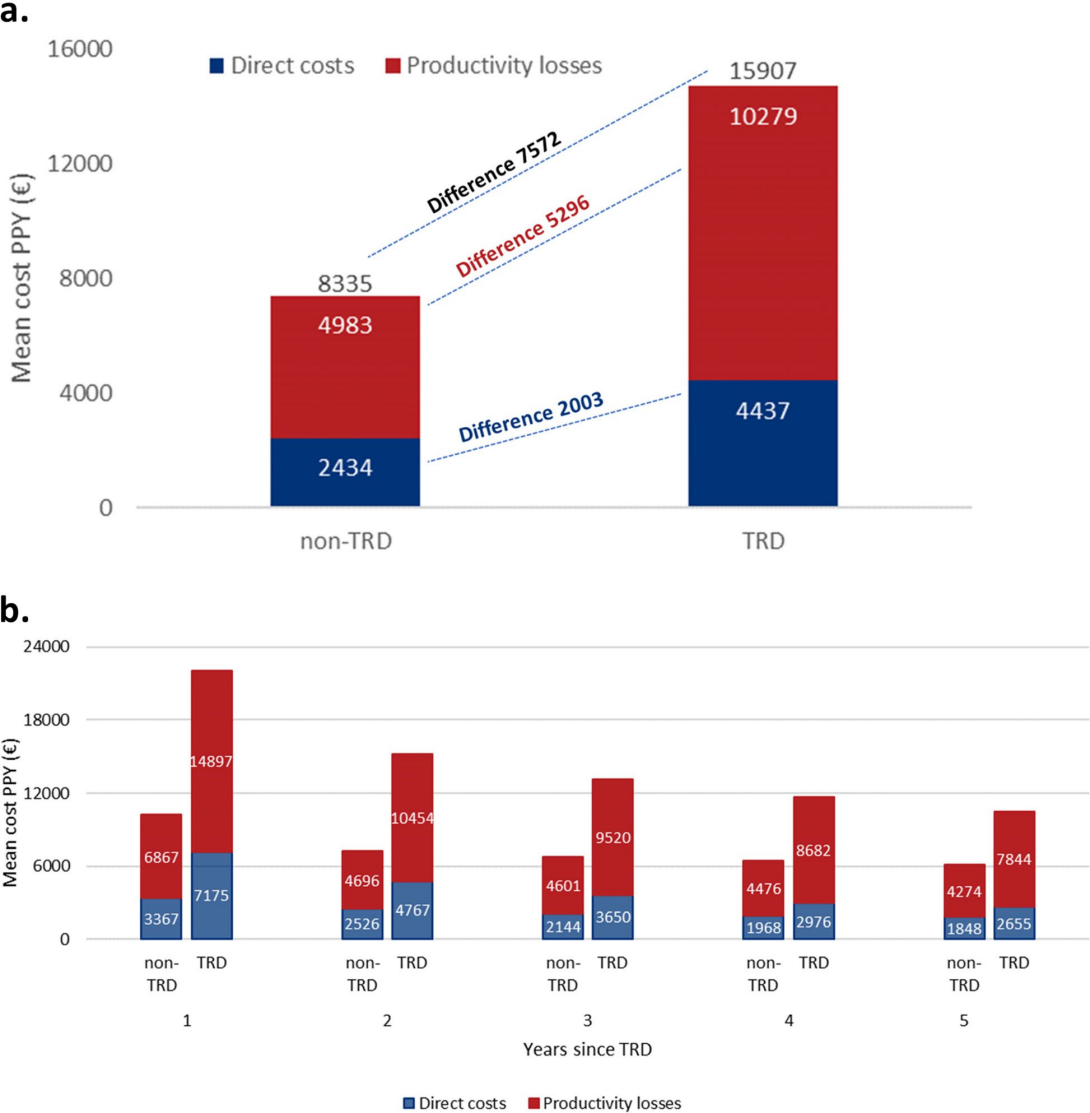
**a** Adjusted mean total costs, and costs divided into direct costs and productivity losses per patient per year (PPY) for treatment-resistant depression (TRD) and non-TRD comparators. Adjusted for: baseline and index period total health care costs, Charlson’s Comorbidity Index, and baseline severity of depression. **b.** Adjusted mean direct costs and productivity losses 1–5 years after treatment-resistant depression (TRD) for persons with TRD and non-TRD as mean of per patient per year (PPY). Adjusted for: baseline and index period total health care costs, Charlson’s Comorbidity Index, and baseline severity of depression

During the 5 years follow-up, persons with TRD had higher health care resource utilization and higher risk for work disability days than comparators (Figure 1), e.g. in outpatient visits (adjusted IRR 1.66, 1.61–1.71) and work disability days (IRR 1.72, 95% CI 1.64–1.80). This resulted in significant adjusted mean cost differences, which were 7572 EUR (95% CI 7215–7929) for total costs, 2003 EUR (1853–2151) for direct costs and 5296 EUR (5042–5550) per patient per year for productivity losses (Fig. 2A). Adjusted mean costs of TRD patients were 1.9-fold, namely 15,907 EUR (95% CI 15646–16,169) per patient per year, compared with 8335 EUR (95% CI 8107–8563) in comparators. Mean cost differences were in similar range in sensitivity analyses with equal censoring for TRD pair members (Supplementary Table 2).

When stratified by years since TRD, the largest difference in adjusted mean total costs was seen during the first year (total costs difference 11,760 EUR, 95% CI 11,314–12,206) and the difference decreased gradually after that (Fig. 2B, Table 2). For the subgroup analyses among those who had been diagnosed with severe depression before matching (*N* = 3894 TRD vs *N* = 1877 non-TRD), the adjusted cost difference was 9206 EUR (8257–1055) per patient per year, mean cost 20,280 (19,680–20,880) for TRD (1.8-fold) and 11,008 EUR (10,291–11,724) for non-TRD (Supplementary Table 3). When matching and start of follow-up was conducted at index antidepressant initiation the mean cost difference between TRD and non-TRD was somewhat larger and the cost levels for both groups were higher than in the main analyses (difference 15,158 EUR, 95% CI 14,368–15,948 per patient per year) (Supplementary Table 4).

**Table 2:** Adjusted mean costs with 95% confidence intervals (CIs) for treatment-resistant depression (TRD) compared with non-TRD and their difference 1–5 years after TRD. Adjusted for: baseline and index period total health care costs, Charlson’s Comorbidity Index, and baseline severity of depression

	non-TRD		TRD		Difference	
	Mean cost	95% CI	Mean cost	95% CI	Mean	95% CI
	Total costs					
Year 1	10,257	9986–10,529	21,928	21,591–22,266	11,760	11,314–12,206
Year 2	7838	7571–8105	15,625	15,297–15,954	7807	7375–8239
Year 3	7724	7456–7993	14,173	13,854–14,493	6443	6019–6868
Year 4	7693	7415–7971	13,273	12,961–13,585	5546	5121–5971
Year 5	7706	7422–7991	12,796	12,480–13,112	5060	4629–5491
	Direct costs					
Year 1	3367	3217–3517	7175	6974–7375	3970	3620–4120
Year 2	2526	2377–2676	4767	4590–4944	2261	2032–2491
Year 3	2144	2014–2273	3650	3494–3806	1508	1310–1705
Year 4	1968	1834–2102	2976	2844–3109	993	808–1178
Year 5	1848	1720–1976	2655	2524–2786	793	613–972
	Hospital costs					
Year 1	1282	1158–1406	2897	2720–3074	1653	1435–1871
Year 2	990	862–1117	1792	1641–1944	814	619–1010
Year 3	813	712–914	1346	1214–1478	535	374–695
Year 4	777	664–890	1059	952–1167	275	125–425
Year 5	728	621–835	927	822–1032	193	49–338
	Outpatient costs					
Year 1	1684	1628–1739	3460	3384–3537	1799	1701–1896
Year 2	1192	1141–1243	2302	2235–2369	1118	1032–1204
Year 3	1048	996–1100	1772	1713–1830	723	645–802
Year 4	973	924–1023	1515	1458–1572	536	460–611
Year 5	930	880–980	1405	1348–1463	469	393–544
	Psychotherapy costs					
Year 1	132	122–141	228	216–240	96	81–112
Year 2	144	134–254	261	247–274	117	100–134
Year 3	110	102–119	189	178–200	79	65–93
Year 4	63	57–60	109	101–118	46	35–57
Year 5	48	42–45	67	60–74	19	9–29
	Drug costs					
Year 1	259	250–267	615	603–627	358	343–372
Year 2	208	199–216	451	439–463	244	229–258
Year 3	186	178–194	381	369–392	195	181–209
Year 4	172	164–180	331	320–342	158	144–172
Year 5	157	149–165	294	283–306	136	123–150
	Productivity loss costs					
Year 1	6867	6677–7058	14,897	14,665–15,128	8026	7723–8336
Year 2	4696	4521–4872	10,454	10,219–10,688	5757	5461–6053
Year 3	4601	4422–4780	9520	9288–9752	4919	4623–5214
Year 4	4476	4296–4655	8682	8454–8910	4206	3914–4498
Year 5	4274	4097–4451	7844	7622–8065	3570	3285–3855
	Sickness absence costs					
Year 1	5062	4921–5204	10,783	10,601–10,965	5716	5483–5949
Year 2	1910	1824–1996	2384	2288–2481	474	343–604
Year 3	1735	1647–1822	2086	1987–2184	351	219–483
Year 4	1531	1446–1617	1843	1745–1941	312	182–443
Year 5	1475	1386–1563	1699	1602–1795	224	93–356
	Disability pension costs					
Year 1	1643	1544–1742	4378	4240–4515	2745	2574–2916
Year 2	3267	3101–3433	8576	8342–8812	5308	5017–5600
Year 3	3650	3469–3831	8444	8203–8686	4787	4481–5093
Year 4	3980	3787–4172	8416	8169–8663	4426	4108–4744
Year 5	4141	3938–4345	8341	8087–8595	4196	3865–4527

**Fig. 3:**
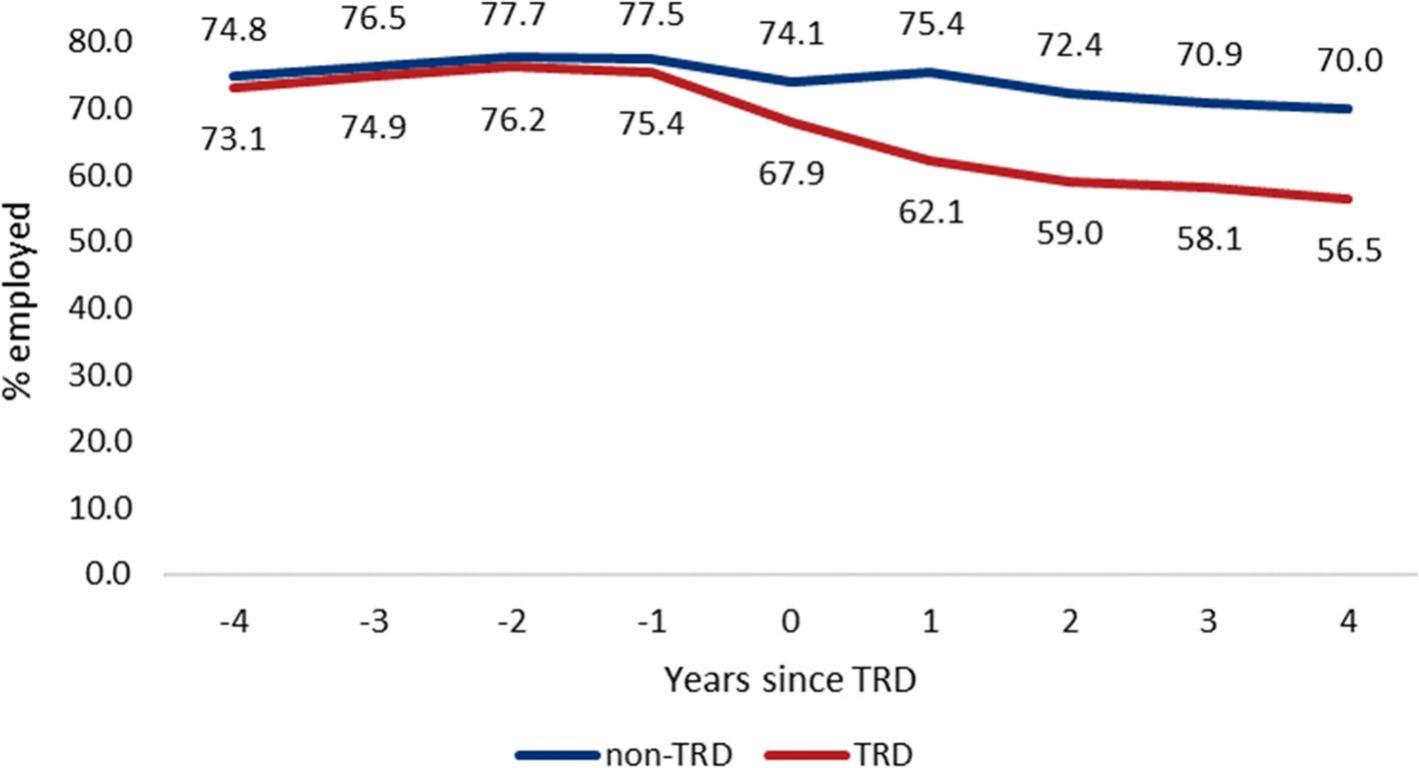
Development of proportion of persons having any income from work as a marker of being employed 4 years before and 4 years after treatment-resistant depression (TRD) in persons with TRD compared with non-TRD persons

Proportion being employed was rather similar 4 years before the matching date between persons with TRD and comparators (73.1% for TRD vs. 74.8% for comparators, Fig. 3). During 4 years before TRD, persons with TRD had slightly lower odds of being employed than comparators (adjusted OR 0.93, 95% CI 0.89–0.97). During the year of TRD, 67.9% of TRD and 74.1% of comparators were employed and the difference increased thereafter. Four years after TRD, 56.5% of persons with TRD and 70.0% of comparators were employed. During the 4 years after TRD, TRD was associated with 36% lower odds of employment than comparators (adjusted OR 0.64, 95% CI 0.62–0.66).

## Discussion

We found TRD to be associated with markedly increased healthcare utilization and higher productivity losses as compared with matched comparison persons without TRD. Both direct healthcare costs and productivity losses were higher in TRD, although the difference was larger for productivity losses, caused by sick leaves and disability pensions. The difference was largest during the first year after TRD and decreased during the 5 years of follow-up, still remaining significant after 5 years. TRD was also associated with decreased odds of being employed.

Higher total costs in persons with TRD are consistent with previous studies which all show a higher cost burden for TRD [14–18]. Some studies have reported equally large differences in direct and indirect costs, [16] others have found a higher difference in direct costs than in indirect costs [18] for TRD compared with non-treatment-resistant patients with depression. In the present study, the cost difference was larger in productivity losses (about 2.1 times higher) than in direct costs (1.8 times higher), and in both TRD and non-TRD, the majority of costs were from productivity losses. These differences between studies likely represent underlying differences in the healthcare, labor market and social security systems between different countries. For example, the direct cost estimates from the US [16] show a higher overall cost level for both TRD and comparators with MDD than in our study, but reported proportional cost differences between TRD and non-TRD are rather similar in the previous studies as in the present study. A recent Swedish study found larger differences between TRD and non-TRD for healthcare utilization, namely hospital days and outpatient visits, than found in our study (Brenner et. al.: Health care utilisation in treatment-resistant depression: a Swedish population-based cohort study, unpublished). Although healthcare systems in Finland and Sweden are rather similar, differences between the studies may stem from the study populations, as we included also persons treated outside of specialized health care (with > 2 weeks sick leave due to depression) whereas the Swedish study included only persons treated in specialized health care.

The follow-up time in our study was 5 years, which is longer than in several previous studies of TRD cost burden, restricting to 2 years [15–17] or less, [18] and they have also started follow-up at index antidepressant prescription whereas our follow-up started at the date when TRD definition was fulfilled (third treatment trial initiation). Our sensitivity analyses starting the follow-up from index antidepressant prescription resulted into a somewhat larger cost difference. This can be at least partly related to the fact that in this design, non-TRD group were selected based on not being defined as TRD during the 2 years definition period whereas in our main analyses, they could serve as comparators until their TRD criteria fulfilment. Both differences in direct costs and productivity losses between TRD and comparator non-TRD patients diminished in 5 years’ time, but especially the difference in productivity losses persisted, being almost two times higher for TRD than for non-TRD also after 5 years. The persisting difference is concerning and highlights the far-reaching, long-term impact of TRD.

Our study also showed a marked difference in employment as persons with TRD were associated with 36% lower odds of being employed than their comparators during 4 years follow-up after TRD. This is in line with previous studies reporting two times higher risk for disability pension, [13] and three-fold larger risk of premature workforce exit [11] and thus, showing a considerably decreased ability to attend working life. Besides high cost burden for the society and additional costs for lost tax payments and lost productivity, decreased participation in working life will also result into lifelong individual economic consequences as retirement-pension is earnings based and lower employment rate will result into a lower retirement-pension.

Subgroup analyses among persons diagnosed with severe form of depression were in line with main analyses regarding higher costs (1.8-fold) associated with TRD. Further, the level of costs associated with severe depression with TRD (total cost 20,280 EUR per patient per year) was higher as compared to the main study cohort for all TRD patients (15,907 EUR) indicating that severity of depression plays an important role in defining high costs. However, TRD was associated still with about two times higher total costs compared with non-TRD comparators which shows that association between TRD and increased costs is not explained by the higher severity of depression. In our previous study, we found that even the fifth treatment line for TRD patients is most commonly antidepressant monotherapy [19]. This is not guideline-driven optimal practice and might be one of the reasons underlying the higher costs in TRD as patients are not receiving the most efficacious treatments available and thus, will use healthcare resources longer and are at an increased risk for permanent work disability which is the main cost driver in TRD. We did not assess the impact of specific comorbidities but adjusted for Charlson comorbidity index. However, a previous study has shown that persons with TRD have more often pre-existing general medical conditions and also subsequent diseases after TRD than their comparators, [25] implying that there may be bidirectional association between TRD and comorbid conditions.

The strengths of this study include nationwide data on persons with TRD and matched comparators with depression. The study cohort included both persons treated in primary and specialized health care during a time period of over 15 years. All residents in Finland are entitled to social and health care services and data on the use of these services is routinely collected. The results of this study are generalizable to healthcare and social security systems resembling that of Finland, i.e. most closely to Nordic countries. Distribution and levels of costs likely differ between different healthcare systems, modified by different access to care and access to social security. Although we comprehensively collected information on all major depression-related health care services, such as inpatient care, specialized outpatient care, medication use and psychotherapies received, some underlying limitations exists. Primary health care utilization data was not available for the study, and some people may have received psychotherapy outside of the state-funded scheme, by covering the costs themselves. We also did not have data on possible failures of psychotherapy and thus, psychotherapies could not be evaluated as possible treatments and treatment lines. In addition, medication costs for the study cohort were only available for psychotropic medications, which limits data describing potential comorbid conditions. However, data on inpatient and specialized outpatient care treatment of these other conditions was included. Productivity losses were based on average gross monthly salary across all sectors and do not take into account possible part-time work. Only persons with residence permit were included in this study and institutionalized persons (e.g. in prisons) were missing.

Baseline severity of depression was adjusted for in all analyses and we conducted subgroup analyses among persons who had received a diagnosis of severe depression by the time of TRD or corresponding matching date. However, the severity level was not available for the entire cohort, due to lack of reporting it (possibly due to technical reasons) in some years especially in the sickness absence register. Definition of TRD as initiation of third treatment trial after two failed trials is similar as in previous studies [7, 10, 13, 26] although the clinical validity of this definition has not been assessed. Data sources did not include data on response to treatment and adherence could only be estimated through dispensing information (i.e. discontinuation of use). Different prescribers may have had different thresholds for prescribing and switching antidepressants although they are expected to follow national guidelines on treatment of depression. Sick leaves and disability pensions are regulated by the social insurance system which harmonizes the decisions between prescribers. In addition, although TRD incurred high costs as compared to non-TRD, these costs are likely underestimates, since we were not able to take into account life-years lost or the effect of work absences, disability and early pension on gross domestic product. The costs related to these phenomena are likely to be much higher than the costs presented here and make the gap between TRD and non-TRD even more pronounced.

## Conclusions

Compared with non-TRD, TRD is associated with about 2-fold cost burden and increased direct costs and productivity losses up to 5 years after TRD. TRD was also associated with reduced likelihood of being employed. These results present persons with TRD as a patient group which would likely benefit from intensified monitoring and multidisciplinary efforts aiming for gaining functional and symptomatic remission and recovery.

## Supplementary Information

**Additional file 1: eFigure 1.** Formation of matched design. AD: antidepressant (or other pharmacological treatment for depression, or ECT), TRD: treatment-resistant depression. **Supplementary Table 1.** Comparison between TRD cases with and without comparison persons, i.e. TRD cases included and excluded from this study. **Supplementary Table 2.** Adjusted mean costs per patient per year with 95% confidence intervals (CIs) for treatment-resistant depression (TRD) compared with non-TRD and their difference 1–5 years after TRD when the follow-up time for the TRD/ non-TRD matched pair was set to equal (censoring both members of the pair when the first member was censored due to any reason). Adjusted for: baseline and index period total health care costs, Charlson’s Comorbidity Index, and baseline severity of depression. Generalized Estimating Equations (GEE) model with gamma distribution and log link. **Supplementary Table 3.** Adjusted mean costs with 95% confidence intervals (CIs) for treatment-resistant depression (TRD) compared with non-TRD and their difference in subgroup of patients with severe depression. Adjusted for: baseline and index period total health care costs, Charlson’s Comorbidity Index, age, gender, hospital district and calendar year. **Supplementary Table 4.** Adjusted mean costs per patient per year with 95% confidence intervals (CIs) for treatment-resistant depression (TRD) compared with non-TRD and their difference 1–5 years after TRD in separate study design where the matching was conducted at index antidepressant initiation and 5-year follow-up started from there (instead of starting from TRD/ matching date). Adjusted for: baseline total health care costs, Charlson’s Comorbidity Index, and baseline severity of depression. Generalized Estimating Equations (GEE) model with gamma distribution and log link.

## Data Availability

The data that support the findings of this study are available from the Finnish government agencies National Institute of Health and Welfare, Social Insurance Institution and Finnish Centre of Pensions, but restrictions apply to the availability of these data, which were used under license for the current study and so are not publicly available. Data are however available from the authors upon reasonable request and with permission of National Institute of Health and Welfare, Social Insurance Institution and Finnish Centre of Pensions.
