# Supplementary material for: Healthcare utilization, costs, and productivity losses in treatment-resistant depression in Finland – a matched cohort study
Source: BMC Psychiatry. 2022 Jul 19;22:484. doi: 10.1186/s12888-022-04115-7 (PMC9297555; doi:10.1186/s12888-022-04115-7)
Supplement: Supplementary file 1 — Additional file 1: eFigure 1. Formation of matched design. AD: antidepressant (or other pharmacological treatment for depression, or ECT), TRD: treatment-resistant depression. Supplementary Table 1. Comparison between TRD cases with and without comparison persons, i.e. TRD cases included and excluded from this study. Supplementary Table 2. Adjusted mean costs per patient per year with 95% confidence intervals (CIs) for treatment-resistant depression (TRD) compared with non-TRD and their difference 1–5 years after TRD when the follow-up time for the TRD/ non-TRD matched pair was set to equal (censoring both members of the pair when the first member was censored due to any reason). Adjusted for: baseline and index period total health care costs, Charlson’s Comorbidity Index, and baseline severity of depression. Generalized Estimating Equations (GEE) model with gamma distribution and log link. Supplementary Table 3. Adjusted mean costs with 95% confidence intervals (CIs) for treatment-resistant depression (TRD) compared with non-TRD and their difference in subgroup of patients with severe depression. Adjusted for: baseline and index period total health care costs, Charlson’s Comorbidity Index, age, gender, hospital district and calendar year. Supplementary Table 4. Adjusted mean costs per patient per year with 95% confidence intervals (CIs) for treatment-resistant depression (TRD) compared with non-TRD and their difference 1–5 years after TRD in separate study design where the matching was conducted at index antidepressant initiation and 5-year follow-up started from there (instead of starting from TRD/ matching date). Adjusted for: baseline total health care costs, Charlson’s Comorbidity Index, and baseline severity of depression. Generalized Estimating Equations (GEE) model with gamma distribution and log link. [file 12888_2022_4115_MOESM1_ESM.docx]

**Supplement**

**eFigure 1.** Formation of matched design. AD: antidepressant (or other pharmacological treatment for depression, or ECT), TRD: treatment-resistant depression.


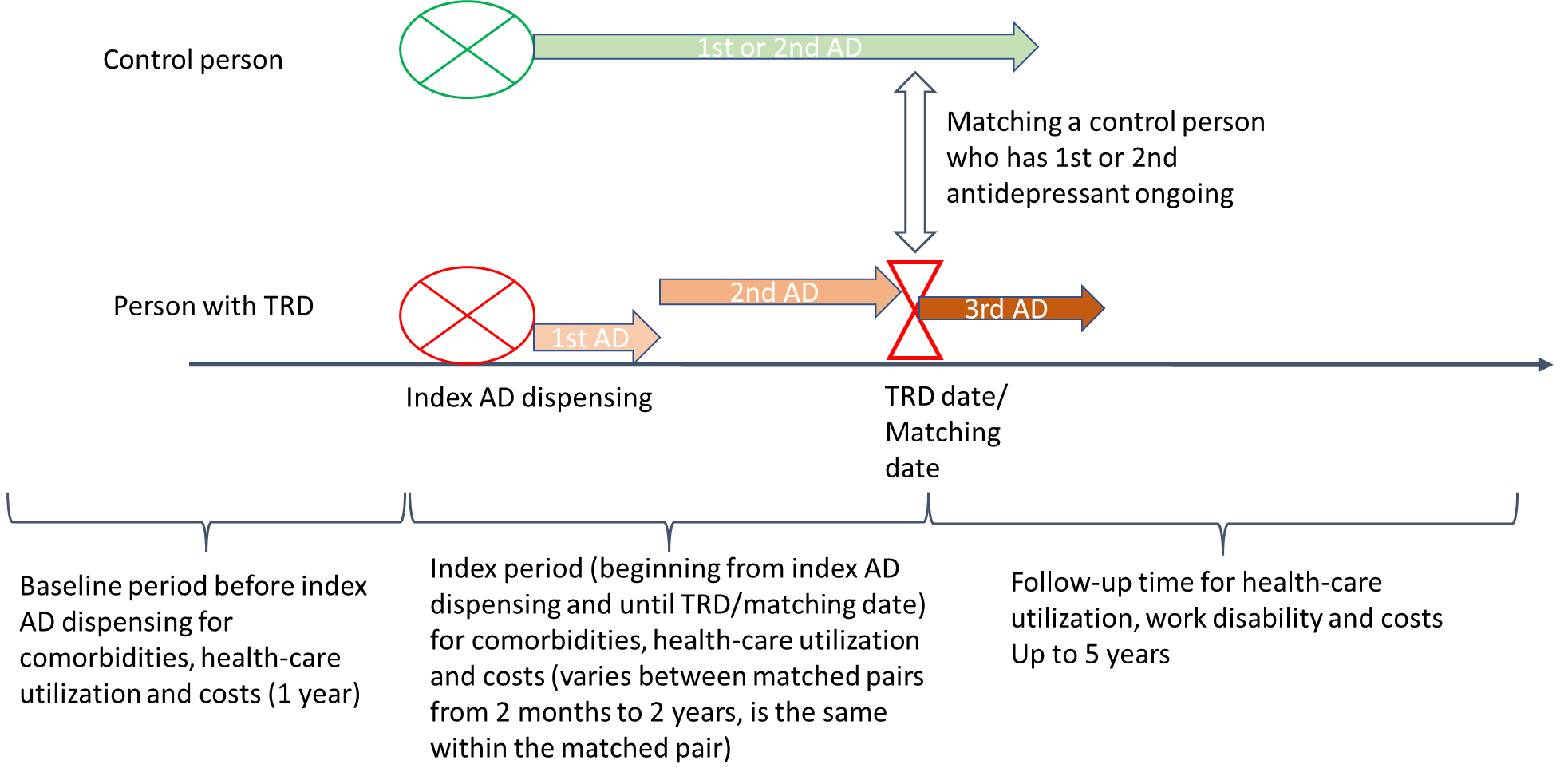


| **Supplementary Table 1**. Comparison between TRD cases with and without comparison persons, i.e. TRD cases included and excluded from this study. | | |
| --- | --- | --- |
|  | TRD cases included N=15405 | TRD cases excluded due to lack of comparison persons N=1248 |
| Male gender, % (N) | 44.5 (556) | 40.0 (6163) |
| Mean age at TRD, (SD) | 38.7 (13.1) | 36.9 (12.8) |
| Mean follow-up time (95% CI), days | 1519 (1510-1528) | 2681 (2597-2763) |
| Mean crude cost per patient per year (95% CI), EUR | 16718 (16400-17036) | 16307 (15192-17421) |

| **Supplementary Table 2.** Adjusted mean costs per patient per year with 95% confidence intervals (CIs) for treatment-resistant depression (TRD) compared with non-TRD and their difference 1-5 years after TRD when the follow-up time for the TRD/ non-TRD matched pair was set to equal (censoring both members of the pair when the first member was censored due to any reason). Adjusted for: baseline and index period total health care costs, Charlson’s Comorbidity Index, and baseline severity of depression. Generalized Estimating Equations (GEE) model with gamma distribution and log link. | | | | | | |
| --- | --- | --- | --- | --- | --- | --- |
|  | **non-TRD** |  | **TRD** |  | **Difference** | |
|  | **Mean cost** | **95% CI** | **Mean cost** | **95% CI** | **Mean** | **95% CI** |
| **Total costs** | | | | |  |  |
| Years 1-5 | 9060 | 8549-9571 | 16777 | 15945-17608 | 7717 | 7212-8222 |
| Year 1 | 11348 | 10726-11970 | 23785 | 22666-24903 | 13191 | 12325-14057 |
| Year 2 | 8555 | 8050-9059 | 16298 | 15500-17096 | 8250 | 7581-8919 |
| Year 3 | 8382 | 7882-8882 | 14477 | 13757-15197 | 6487 | 5873-7101 |
| Year 4 | 8136 | 7636-8636 | 13451 | 12773-14129 | 4696 | 4269-5124 |
| Year 5 | 8020 | 7520-8519 | 12830 | 12172-13489 | 4264 | 3828-4701 |
| **Direct costs** | | | | | | |
| Years 1-5 | 4012 | 3060-4963 | 7184 | 5507-8862 | 3173 | 2410-3935 |
| **Productivity losses** | | | | | | |
| Years 1-5 | 4870 | 4664-5075 | 9652 | 9371-9934 | 4782 | 4513-5052 |

| **Supplementary Table 3.** Adjusted mean costs with 95% confidence intervals (CIs) for treatment-resistant depression (TRD) compared with non-TRD and their difference in subgroup of patients with severe depression. Adjusted for: baseline and index period total health care costs, Charlson’s Comorbidity Index, age, gender, hospital district and calendar year. | | | |
| --- | --- | --- | --- |
|  | **Mean** | **95% Confidence Interval** | |
| **Total costs** | | |  |
| non-TRD | 11008 | 10291-11724  19680-20880  8257-10155 | |
| TRD | 20280 |  |  |
| difference | 9206 |  |  |
| **Direct costs** | | |  |
| non-TRD | 4160 | 3791-4530  6567-7175  2234-3188 | |
| TRD | 6871 |  |  |
| difference | 2711 |  |  |
| **Productivity losses** | |  |  |
| non-TRD | 5529 | 5062-5996  11158-11975  5414-6661 | |
| TRD | 11566 |  |  |
| difference | 6038 |  |  |

| **Supplementary Table 4.** Adjusted mean costs per patient per year with 95% confidence intervals (CIs) for treatment-resistant depression (TRD) compared with non-TRD and their difference 1-5 years after TRD in separate study design where the matching was conducted at index antidepressant initiation and 5-year follow-up started from there (instead of starting from TRD/ matching date). Adjusted for: baseline total health care costs, Charlson’s Comorbidity Index, and baseline severity of depression. Generalized Estimating Equations (GEE) model with gamma distribution and log link. | | | | | | |
| --- | --- | --- | --- | --- | --- | --- |
|  | **non-TRD N=17912** |  | **TRD N=17912** |  | **Difference** | |
|  | **Mean cost** | **95% CI** | **Mean cost** | **95% CI** | **Mean** | **95% CI** |
| **Total costs** | | | | |  |  |
| Years 1-5 | 11253 | 10736-11769 | 26411 | 25222-27600 | 15158 | 14368-15948 |
| Year 1 | 16359 | 15609-17110 | 35901 | 34257-37545 | 19542 | 18532-20551 |
| Year 2 | 10338 | 9826-10850 | 28544 | 27225-29863 | 18240 | 17268-19222 |
| Year 3 | 9713 | 9226-10200 | 23556 | 22456-24657 | 13843 | 13038-14649 |
| Year 4 | 9659 | 9174-10144 | 21709 | 20687-22731 | 11988 | 11245-12732 |
| Year 5 | 9596 | 9109-10082 | 20616 | 19635-21597 | 11058 | 10327-11789 |
| **Direct costs** | | | | | | |
| Years 1-5 | 3190 | 2948-3432 | 8027 | 7451-8604 | 4837 | 4458-5217 |
| **Productivity losses** | | | | | | |
| Years 1-5 | 7361 | 7026-7696 | 16526 | 15796-17256 | 9165 | 8660-9670 |
